# Functional divergence of MADS-box genes and the dual regulatory role of a long noncoding RNA underlie sex determination in *Ginkgo biloba*

**DOI:** 10.1186/s12870-026-09503-9

**Published:** 2026-07-10

**Authors:** Huaitong Wu, Baoyu Zheng, Kaiyuan Liu, Tongming Yin, Suyun Wei

**Affiliations:** 1 State Key Laboratory of Tree Genetics and Breeding, Key Laboratory of Tree Genetics and Biotechnology of Educational Department of China, Key Laboratory of Tree Genetics and Silvicultural Sciences of Jiangsu Province, Co-Innovation Center for Sustainable Forestry in Southern China, Nanjing Forestry University, Nanjing, 210037 China; 2 College of Information Science and Technology & Artificial Intelligence, Nanjing Forestry University, Nanjing, 210037 China

**Keywords:** *Ginkgo biloba*, Sex determination, MADS-box genes, Long noncoding RNAs, Sex-determining region

## Abstract

**Background:**

*Ginkgo biloba* is a dioecious gymnosperm that has remained morphologically stable for over 200 million years, offering a unique system for studying early plant reproductive evolution. However, the molecular mechanisms underlying its sex determination remain largely unknown due to its large genome and the lack of genetic transformation tools. The purpose of this study was to precisely map the sex-determining region and decipher the gene regulatory network governing sexual differentiation in this ancient species.

**Results:**

Through bulked segregant analysis sequencing (BSA-seq) and transcriptomic profiling of male and female pools, we mapped a sex-determining region (SDR) to a nine-megabase interval on chromosome two. Within this region, a B-class MADS-box transcription factor, *GbAP3a*, was identified as a core candidate promoting male development. It exhibits strict male-specific expression in microstrobili, and its sequence and function are highly conserved due to tandem duplication and strong purifying selection. We also characterized a male-specific long noncoding RNA, *GbLINC-CNR1* (Cellular Negative Regulator 1), located within this region. This long noncoding RNA regulates sex differentiation via two parallel pathways: it represses the MADS transcription factor *GbAGL32a* to abort ovule development, and it acts as a competing endogenous RNA for microRNA 159 to prevent the degradation of *GbMYB33* transcripts, thereby sustaining pollen development.

**Conclusions:**

Sex determination in *Ginkgo* is orchestrated by the functional divergence of two MADS-box genes, tightly integrated with dual regulatory inputs from a sex-linked long noncoding RNA. These findings provide mechanistic insights into the evolution of sex chromosomes and reproductive strategies in gymnosperms, offering potential molecular targets for early sex identification and marker-assisted breeding in dioecious trees.

**Supplementary Information:**

The online version contains supplementary material available at https://doi.org/10.1186/s12870-026-09503-9.

## Introduction

As a dioecious gymnosperm that has remained morphologically stable for over 200 million years, *Ginkgo biloba* offers a window into ancient plant reproductive strategies [1, 2]. Its strictly separate male and female individuals, large genome, and long generation time make it both challenging and valuable for studying how sex chromosomes evolve and how sexual dimorphism arises [2, 3]. In plants, the transition from hermaphroditism to dioecy is often linked to the formation of sex chromosomes. According to the Charlesworth model, this involves the accumulation of mutations that suppress one sexual function, followed by recombination suppression around these loci, eventually creating a distinct SDR [4, 5]. Over the past two decades, SDRs have been identified in several dioecious species, including poplar, kiwifruit, and persimmon, revealing diverse molecular mechanisms [6–9].

The ABCDE model of floral organ identity offers a crucial genetic framework for understanding sexual differentiation [10–12]. In this model, B-, C-, and D-class genes collectively determine the formation of stamens, carpels, and ovules, respectively [13–15]. The majority of ABCDE model genes belong to the MIKC-type MADS-box family, whose protein products contain four sequential domains: the MADS (M) domain, the intervening (I) domain, the keratin-like (K) domain, and the C-terminal (C) domain [16, 17]. MADS-box transcription factors recognize and bind CArG box cis-elements in gene promoters through their highly conserved MADS domain [18], thereby activating downstream target genes during reproductive development [19]. Both B- and C-class MADS-box genes have been implicated in sex determination processes across multiple dioecious plant species [20, 21]. The B-class MADS-box genes *APETALA3* (*AP3*) and *PISTILLATA* (*PI*) form a central regulatory module specifying petal and stamen identity, and this functional module demonstrates remarkable evolutionary conservation across the angiosperm lineage, from eudicot model organisms to monocots and woody perennials [22–26]. In Norway spruce (*Picea abies*), B-function MADS-box genes (*DAL11*, *DAL12*, and *DAL13*) are phylogenetically related to angiosperm B-class genes and are specifically expressed in developing pollen cones, suggesting that the B-gene function in specifying male reproductive organ identity has been conserved between conifers and angiosperms [27]. Furthermore, functional studies have demonstrated that ectopic expression of these spruce B-type genes in Arabidopsis can induce homeotic changes, including the formation of ectopic anthers [28]. These findings support the view that a “BC system” for specifying male versus female reproductive organs existed in the common ancestor of seed plants, with B-class genes playing a central role in promoting male reproductive development long before the origin of flowers.

Beyond protein-coding genes, long noncoding RNAs (lncRNAs) have emerged as important players in plant reproduction. They can function through diverse mechanisms, including direct interactions with proteins, DNA, or other RNAs, thereby participating in floral organ identity transitions, meiosis progression, anther development, and pollen formation [29–32]. In poplar, for instance, the Y chromosome-specific gene MSL produces lncRNAs that actively promote male sex determination [9, 33]. Additionally, lncRNAs can act as endogenous target mimics (eTMs) to modulate microRNA (miRNA) activity by preventing miRNA binding to their target genes, thus indirectly regulating downstream gene expression and influencing sex-related developmental pathways [34–36]. This eTMs mechanism represents a key route for lncRNA-mediated regulation of plant sex determination. Despite these advances, the specific role of lncRNAs in gymnosperm sex determination remains largely unexplored.

Given its enormous genome and the lack of genetic transformation tools, identifying sex-determining genes in *Ginkgo* has been slow. Earlier studies placed the SDR on chromosome 2, but with limited resolution [3, 37]. Here, we revisit this problem using a combination of BSAseq, RNA-seq, and lncRNA sequencing on male and female individuals from a natural population. Our goal was to refine the SDR location, identify key candidate genes, and unravel the regulatory network—particularly the role of lncRNAs—that governs sexual differentiation in this ancient lineage.

## Results

### Morphological differences and developmental timing between male and female reproductive organs

**Fig. 1:**
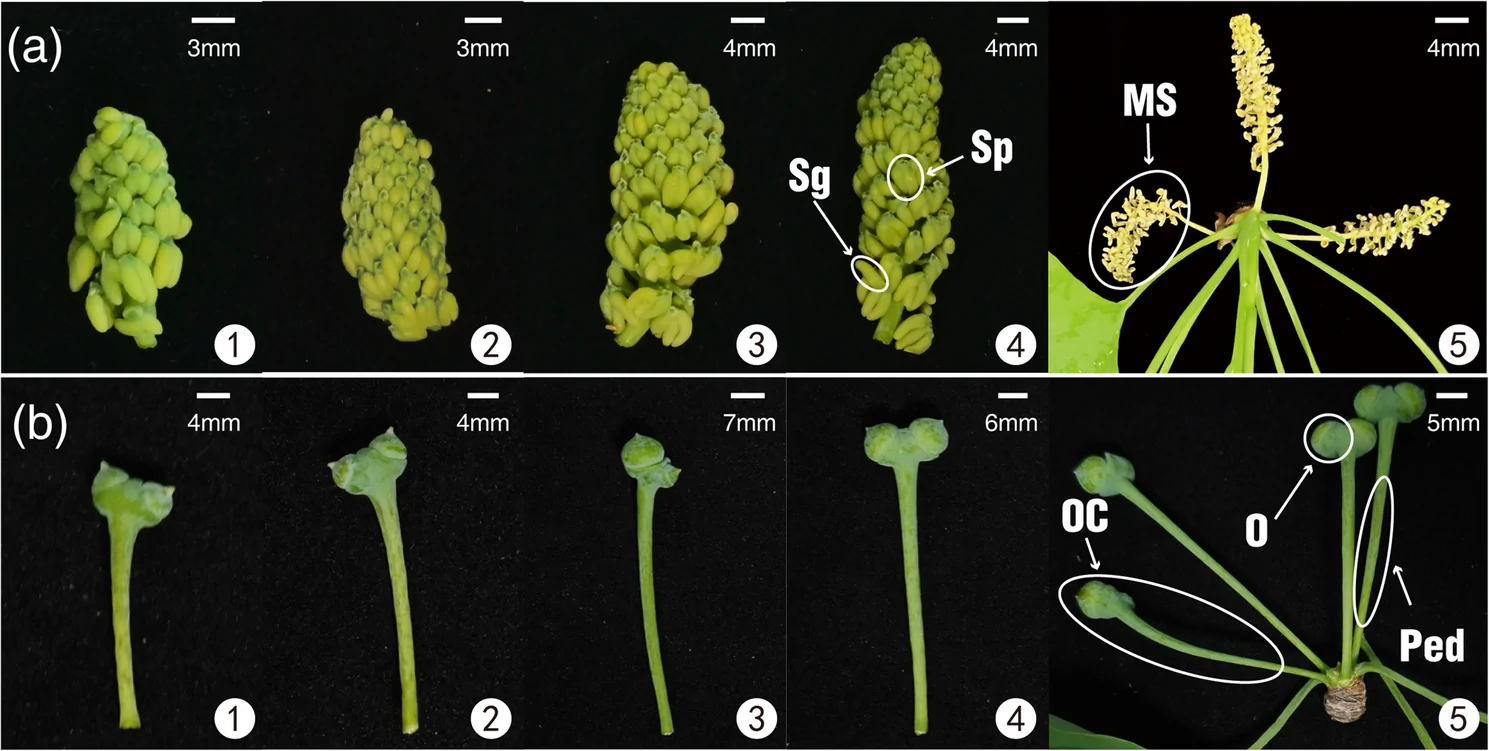
Morphological development of male and female reproductive organs in *Ginkgo biloba. ***a** Development of the male microstrobilus. Images 1 to 4 illustrate the progressive morphological transitions from primordium differentiation to maturation, while image 5 shows the mature microstrobili arranged on a short shoot. **b** Morphogenesis of the female ovulate complex. Images 1 to 4 depict the peduncle elongation and apical ovule differentiation, and image 5 presents the mature ovule complex with characteristic dichotomous branching. MS, microstrobilus; Sp, microsporophyll; Sg, microsporangia; OC, ovule complex; O, ovule; Ped, peduncle

As a dioecious species, *G biloba* shows clear dimorphism not only in the structure of its reproductive organs but also in when they develop. Male reproductive organs (microstrobili) begin differentiating in the summer before flowering. By late autumn, the primordia of microsporophylls are already formed within the bud and enter winter dormancy. After bud break in spring, development resumes quickly, with microsporogenesis completed within four to six weeks. Each male bud typically produces two to eight pedicellate microstrobili spirally arranged on a short axis. A mature microstrobilus contains 60–80 microsporophylls, each bearing two ellipsoidal microsporangia on the abaxial side. These dehisce to release wind-dispersed pollen (Fig. 1a).

In contrast, female reproductive structures (ovulate complexes) initiate differentiation only after bud break in spring. This delay likely protects the exposed ovules from early spring frosts. The peduncle apex undergoes dichotomous branching, a primitive feature, with each branch tip developing into a single ovule. At pollination, the peduncle elongates to 3–5 cm, forming a distinctive bifurcating structure. Mature ovules have a fleshy outer integument that secretes a pollination drop, a sclerified inner integument enclosing the nucellus, and a prominent micropyle that guides pollen. The pollination drop, rich in sugars and amino acids, captures pollen and promotes germination (Fig. 1b).

### Mapping the sex-determining region and identifying candidate genes

To precisely delineate the SDR in *G. biloba*, we employed whole-genome variant detection through BSA-seq. High-throughput whole-genome resequencing was performed on pooled male and female samples, generating a substantial dataset of 1,158.52 Gb of raw data, with an average sequencing depth of approximately 80×. Following stringent quality control filtering, 96.5% of the data remained as high-quality clean reads, with an impressive average Q30 value of 91.51% (Supplementary Table 1). These clean reads were mapped to the *Ginkgo* reference genome with an alignment rate of 91.23%, enabling the identification of 67,196,312 single nucleotide polymorphisms (SNPs) and 4,763,312 insertion/deletion (InDel) variants. Among these variants, 98.5% of SNPs and 77.1% of InDels were successfully anchored to the 12 chromosomes of *G. biloba*, with an average SNP density of approximately 7 variants per kilobase (Supplementary Table 2). Notably, these variants were distributed unevenly across the genome, with markedly reduced density at the terminal regions of chromosomes (Supplementary Fig. 1). Functional annotation revealed that over 99% of variants resided in intergenic or other noncoding regions. Although coding region variants accounted for less than 0.2% of the total, they included potentially function-altering mutations: 45,641 nonsynonymous SNPs predicted to cause amino acid substitutions, 3,226 frameshift InDels likely disrupting protein translation, 1,112 stop-gain mutations, 277 codon losses, and 403 variants affecting splice sites (Supplementary Table 3).

**Fig. 2:**
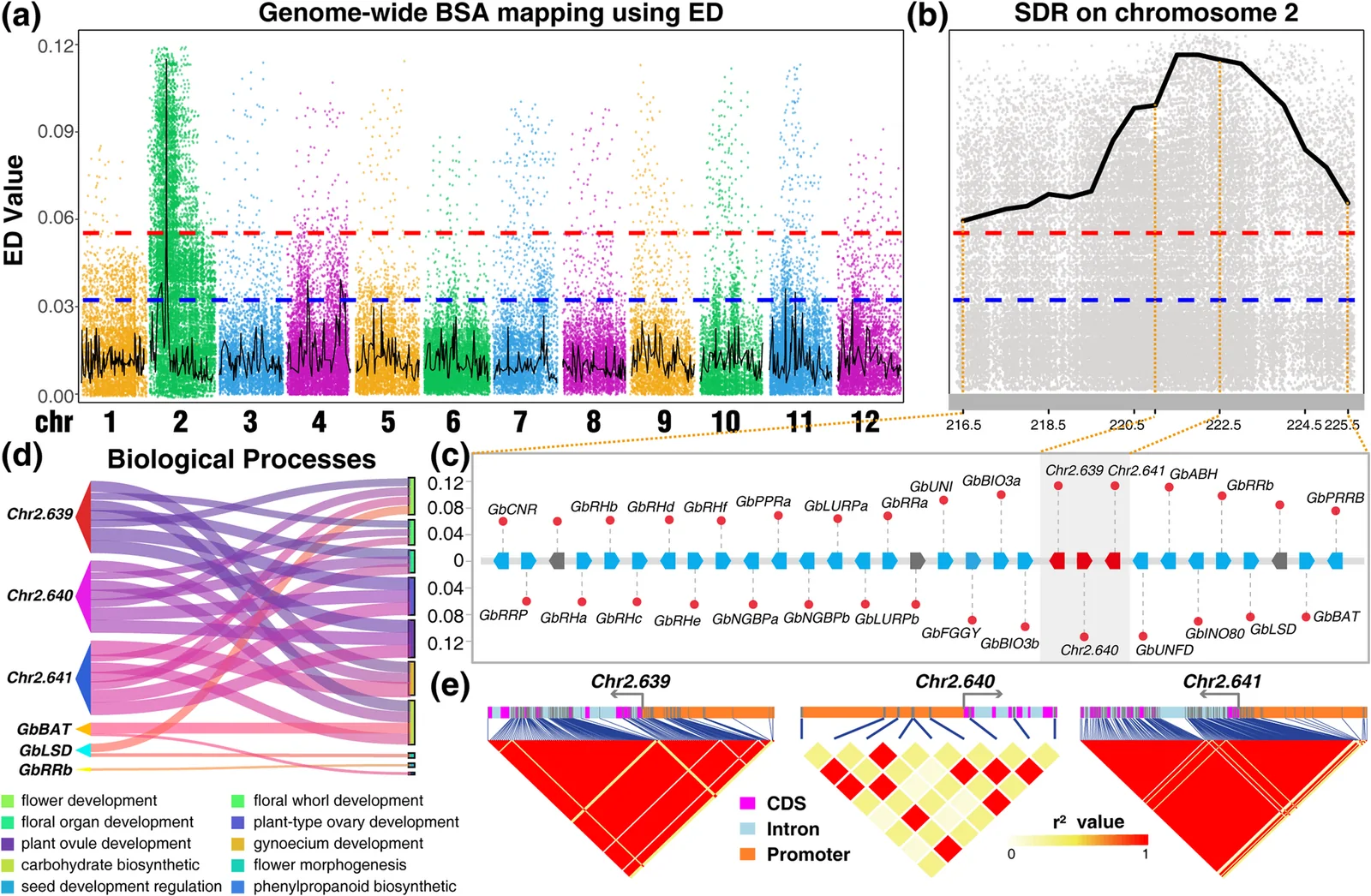
Identification of the SDR in *G. biloba* using BSA and characterization of candidate genes. **a** Genome-wide BSA association analysis based on Euclidean distance (ED) values. Colored dots represent ED values of individual SNPs. The black curve indicates the sliding window fitting (5 Mb window size, 500 kb step size). The red and blue dashed lines denote the 99% and 95% confidence thresholds, respectively. **b** Magnified view of the significant association signal within the 216.5-225.5 Mb interval on chromosome 2, which defines this 9 Mb interval as the SDR in G. biloba. **c** Gene arrangement within the SDR. Pentagons represent gene models, with red denoting the three key candidate genes (*chr2.639*, *chr2.640*, and *chr2.641*) in the core peak region, blue indicating functionally annotated genes, and gray representing genes of unknown function. **d** Gene Ontology (GO) enrichment analysis of key candidate genes. Connecting ribbons link genes to their enriched GO biological processes, and ribbon width reflects enrichment significance. **e** Linkage disequilibrium (LD) heatmap of the key gene region within the SDR. LD coefficients (r² values) were calculated using high-quality SNP genotyping data obtained from all resequenced individuals (30 females and 30 males). Genomic features are annotated as CDS (purple), introns (blue), and promoters (orange). Heatmap color intensity represents pairwise LD strength, with red areas showing high LD blocks within *chr2.639* and *chr2.641*

Based on Euclidean Distance (ED) analysis of allele frequency differences between male and female pools, we identified a significant association peak spanning 9 Mb within the 216.5-225.5 Mb interval on chromosome 2, which we defined as the SDR in *G. biloba* (Fig. 2a, b; Supplementary Table 4). Within it, 85.1% of SNPs were heterozygous in males but homozygous in females, supporting an XX/XY sex-determination system (Supplementary Fig. 2). Gene Ontology (GO) enrichment analysis of the 31 genes within the SDR revealed that three genes (*chr2.639*, *chr2.640*, and *chr2.641*) located at the core ED peak, were significantly enriched in key biological processes related to flower development, floral whorl development, and floral organ development (Fig. 2d; Supplementary Fig. 3; Supplementary Table 5). Functional annotation and genomic structural analysis indicated that all three genes encode MADS-box transcription factors and are arranged as tandem duplications within the SDR (Fig. 2c). Linkage disequilibrium (LD) analysis based on SNP genotyping data from all resequenced individuals (30 females and 30 males) revealed a strong LD signal between SNPs located within *chr2.639* and *chr2.641* in the SDR, indicating strong recombination suppression across this interval and implicating these genes as conserved, potential core components of sex determination in *G. biloba* (Fig. 2e).

### Expression patterns of floral identity genes suggest a simplified BCDE model in *G. biloba*

**Fig. 3:**
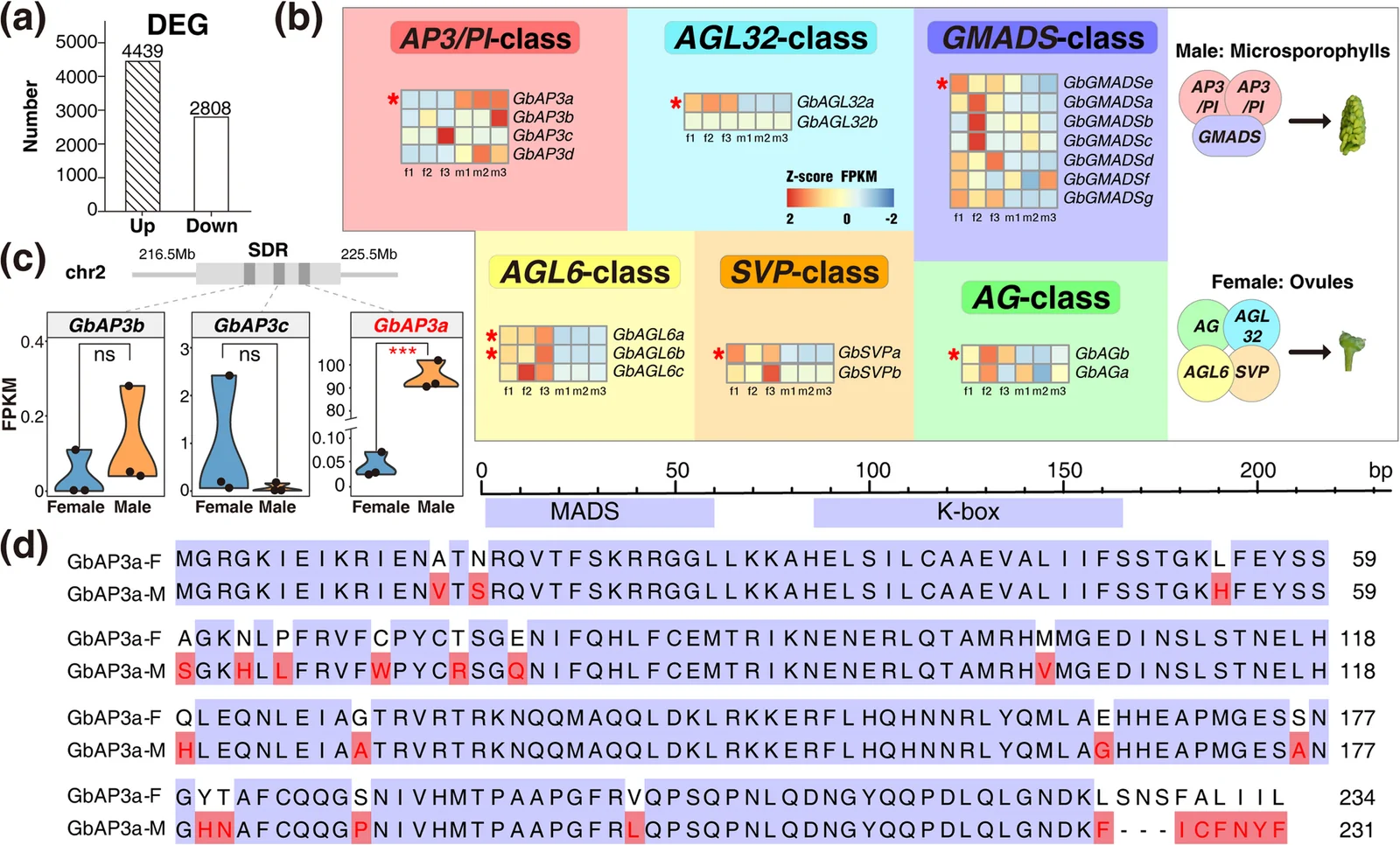
Identification and expression analysis of sex-determining genes in *G. biloba. ***a** The number of differentially expressed genes (DEGs) between female and male *G. biloba* individuals. **b** Expression patterns of MIKC-type MADS-box genes involved in reproductive development in *G. biloba*. The heatmap shows the relative expression levels of these genes between male and female samples. Red indicates high expression, blue indicates low expression, and red asterisks denote differentially expressed genes. **c** Expression profiles of candidate genes from the SDR in male and female tissues. The violin plots display the FPKM (Fragments Per Kilobase of transcript per Million mapped reads) values for each gene. Asterisks indicate statistically highly significant differences in expression levels (***P < 0.001, two-tailed Student’s t-test), and ns indicates no significant difference. **d** Alignment of GbAP3a protein sequences derived from male and female individuals. The upper scale indicates the positions of the conserved MADS domain and K-box domain. Amino acid mutation sites specific to the male GbAP3a sequence are marked in red

To identify key genes involved in sex determination and construct a regulatory model for reproductive organ development in *G.biloba*, transcriptome sequencing was performed on mixed pools of male and female samples. A total of 67.08 Gb of high-quality data was generated, with Q30 value exceeding 94.04%, and 447 million clean reads were retained after quality control (Supplementary Table 6). Genome-wide analysis identified 7,247 DEGs, of which 2,808 genes (38.7%) were downregulated and 4,439 genes (61.3%) were upregulated in male individuals compared to females (Fig. 3a, Supplementary Fig. 4). GO and KEGG enrichment analyses showed that the DEGs were predominantly associated with floral development and secondary metabolism, including significant enrichment in biological processes such as pollen development and in flavonoid and phenylpropanoid biosynthesis pathways, which are closely linked to reproductive organ differentiation (Supplementary Fig. 5).

To systematically characterize the MADS-box gene family in *G. biloba*, we identified 29 MADS-box genes, including 6 type I and 23 type II members (Supplementary Table 7). Structural analysis revealed that type II members retained complete MIKC domains, whereas type I members generally lacked the K domain, a difference that may underlie divergence in their dimerization capacity and functional roles. These MADS-box genes are distributed across eight chromosomes, with some arranged in clusters, suggesting potential local duplication events (Supplementary Fig. 6). Evolutionary analysis indicated that expansion of the *G. biloba* MADS-box family primarily resulted from small-scale tandem and proximal duplications rather than whole-genome duplication events. Notably, *chr2.641* and its paralog *chr2.640* form a tandem duplication pair with a low *Ka*/*Ks* ratio (0.23), indicating strong purifying selection (Supplementary Table 8).

In the ABCDE model of floral organ identity, with the exception of the A-class gene *APETALA2* (AP2), which belongs to the AP2/ERF family, the vast majority of floral organ identity genes are II-type MADS-box genes. Using the phylogenetic framework established by Chen et al. (2017) [38], we reconstructed a maximum-likelihood tree incorporating the 23 MIKC-type genes together with reference sequences from representative gymnosperms and angiosperms (Supplementary Fig. 7). The resulting phylogeny resolved 22 of these genes into eight distinct MIKC^C^ clades: AG (2 members), AGL32 (2), AGL6 (3), AP3/PI (4), GMADS (7), SVP (2), SOC1 (1), and ANR1 (1), with one additional MIKC* gene (*chr12.1550*) also identified (Supplementary Table 9). Following clade assignments, each gene was designated with the prefix “Gb” followed by the clade name and an alphabetical suffix (e.g., GbAGa; Supplementary Table 9). Notably, three MADS-box genes located within SDR—*chr2.639*, *chr2.640*, and *chr2.641*—were named *GbAP3b*, *GbAP3c*, and *GbAP3a*, respectively.

Transcriptomic profiling of male and female strobili revealed four distinct expression patterns among the MIKC^C^ genes. The B-class gene *GbAP3a* showed highly significant and specific upregulation in male strobili but was nearly undetectable in female strobili (FPKM = 98.1 in males versus near zero in females; Fig. 3b and c), positioning it as a core factor initiating male reproductive organ identity. By contrast, the C-class gene *GbAGb*, two AGL6-clade genes (*GbAGL6a* and *GbAGL6b*), one AGL32-clade gene (*GbAGL32a*), and two SVP-clade genes (*GbSVPa* and *GbSVPb*) were all significantly upregulated in female strobili (Fig. 3b). Given that gymnosperms naturally lack E-class (SEP) genes, this coordinated upregulation suggests that these genes may form alternative protein complexes to drive female reproductive organ specification. *GbAGL6c* and the five GMADS members exhibited no significant expression differences between sexes, suggesting housekeeping functions in reproductive development, whereas the remaining MIKC-type members members showed extremely low or undetectable expression in both strobilus types (Supplementary Table 10).

Further analysis integrating DEGs within the SDR identified *GbAP3a* as the prime candidate for sex determination: comparison of *GbAP3a* sequences between sexes revealed multiple nonsynonymous mutations in males, some located in the highly conserved MADS domain (Fig. 3d), and its promoter contains CArG box cis-regulatory elements that could be bound by MADS-box proteins, potentially forming regulatory complexes (Supplementary Fig. 8; Supplementary Table 11). As a type II member, *GbAP3a* possesses a complete K domain, which likely enhances its interaction with other MADS proteins and facilitates its role in male reproductive development. This hypothesis is substantiated by the phylogenetic clustering of *GbAP3a* with canonical B-class genes (Supplementary Fig. 7) and the extensive functional conservation of AP3 orthologs in stamen specification observed across diverse angiosperms and gymnosperms [27, 39, 40].

Collectively, these results demonstrate that sex differentiation in *Ginkgo* is governed by a dynamic balance between the male-promoting factor (*GbAP3a*, a B-class MADS-box gene) and a female core complex comprising the C-class gene *GbAGb*, AGL6-clade genes (*GbAGL6a/b*), the B-sister gene *GbAGL32a*, and SVP-clade genes (*GbSVPa/b*). This expression architecture supports the gymnosperm “BC system” hypothesis, in which B-class genes promote male reproductive development, while C-class genes, either alone or in combination with other MADS-box proteins, form multimeric complexes to specify female reproductive organ identity in the absence of angiosperm-specific SEP (E-class) proteins [39, 41].

### The long noncoding RNA regulates sex differentiation in *G. biloba* through dual molecular mechanisms

To explore lncRNA functions, we sequenced lncRNA transcriptomes from male and female samples (Supplementary Table 12). Each sample yielded over 753 million clean reads, totaling 112.98 GB, with Q30 values between 90.1% and 94.0%. After stringent filtering, we identified 1,001 lncRNAs, most (93.4%) intergenic (Supplementary Fig. 9).

**Fig. 4:**
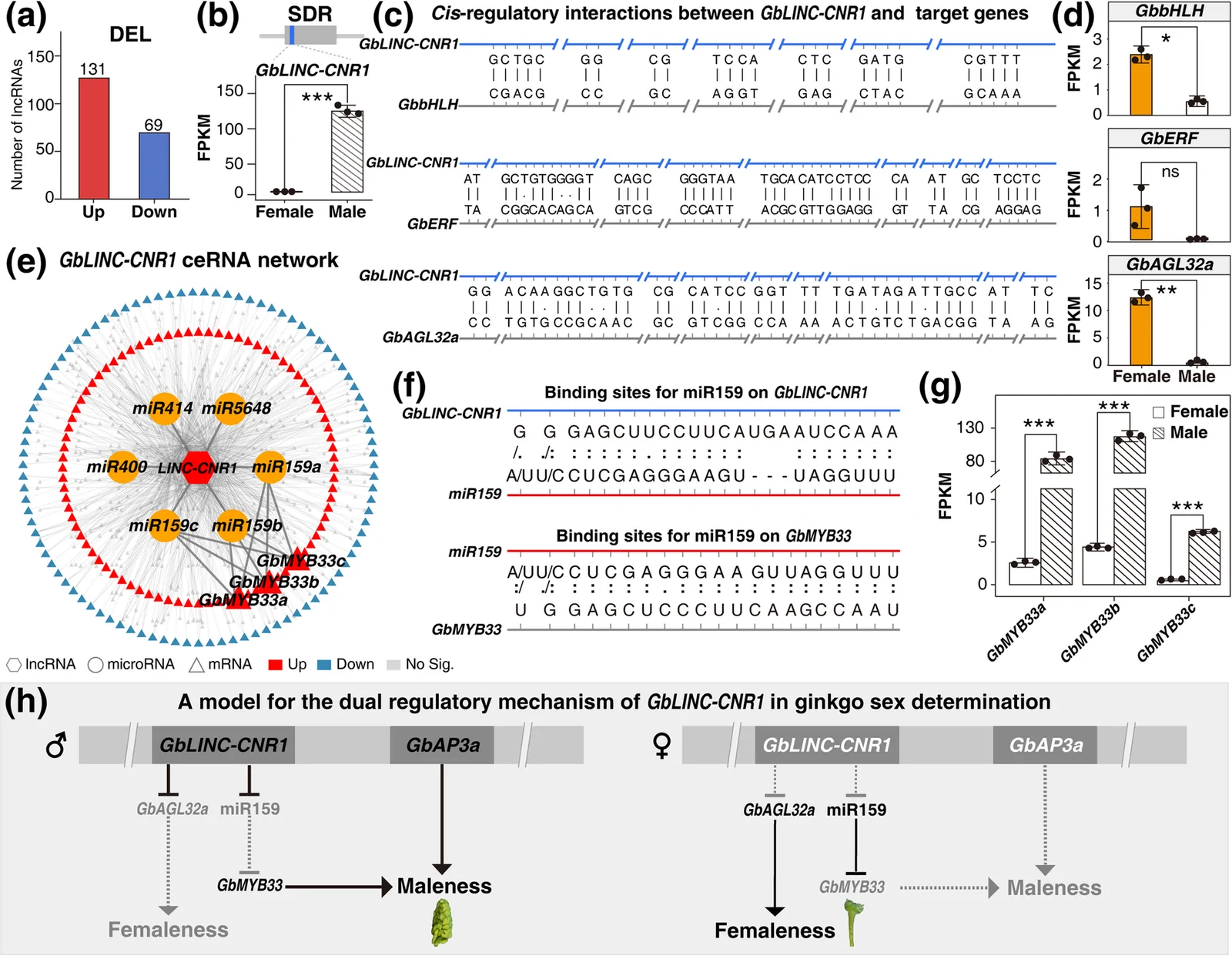
The dual regulatory mechanism of *GbLINC-CNR1* in the sex differentiation of *G. biloba. ***a** Number of upregulated and downregulated DELs between female and male samples. Red and blue bars represent the number of genes upregulated and downregulated in males respectively. **b** Expression profile of *GbLINC-CNR1* (blue track) within the *Ginkgo* sex-determining region (dark gray area) in female and male samples. **c** Predicted base-pairing interactions between GbLINC-CNR1 and its target mRNAs. Vertical bars denote standard Watson-Crick base pairs, while dots indicate noncanonical or wobble base pairs. **d** Expression patterns of the regulated target genes of *GbLINC-CNR1* in female and male individuals. **e** The ceRNA regulatory network centered on *GbLINC-CNR1*. Hexagons, circles, and triangles depict lncRNAs, miRNAs, and mRNAs respectively. Red, blue, and gray fills indicate upregulation, downregulation, and no significant difference in males. **f** Predicted miR159 binding sites on *GbLINC-CNR1* (top) and *GbMYB33* (bottom). **g** Expression patterns of three *GbMYB33* genes within the ceRNA network. **h)** Schematic model of *GbLINC-CNR1* dual-pathway regulation in sex differentiation. Arrows indicate activation, and blunt-ended arrows denote inhibition. Expression values are the mean ± standard error from three biological replicates. Significant differences between female and male individuals are indicated by asterisks where *p < 0.05, **p < 0.005, and ***p < 0.001 according to a two-tailed Student’s t-test. The term ns denotes a non-significant difference

Differential expression analysis identified 200 significantly differentially expressed lncRNAs (DELs), including 131 upregulated and 69 downregulated in males (Fig. 4a, Supplementary Fig. 10). Motif prediction for these DELs revealed four enriched sequences, with three (ACACASAG, AGAGRAG, CTCKSC) identified as putative transcription factor binding sites (Supplementary Table 13). Notably, an intergenic lncRNA located within the sex-determining region, named *GbLINC-CNR1* (Cellular Negative Regulator 1), showed remarkably male-specific expression: it was abundant in male catkins but barely detectable in female tissues (Fig. 4a, b; Supplementary Fig. 11). Functionally, *GbLINC-CNR1* appeared to repress the AGL32-class MADS-box gene *GbAGL32a*, the bHLH gene *GbbHLH*, and the ethylene response factor *GbERF* (Fig. 4c). Since *GbAGL32a* is crucial for ovule development and is barely expressed in males (Fig. 4d), *GbLINC-CNR1* likely suppresses female organogenesis in male trees.

*GbLINC-CNR1* may also act as a competing endogenous RNA (ceRNA). We predicted that it could interact with several miRNAs, including miR159, and influence 713 target mRNAs (Fig. 4e). Three *MYB33* genes (*GbMYB3a*, *GbMYB3b*, *GbMYB3c*) are predicted targets of miR159 and were upregulated in males (Fig. 4f, g). This is consistent with *GbLINC-CNR1* acting as a molecular sponge for miR159, protecting *GbMYB33* transcripts from degradation and thereby promoting pollen development. Supporting this model, we found that *GbMYB33a* and *GbMYB33b* proteins localize to both the nucleus and cytoplasm, suggesting their mRNAs could be accessible to cytoplasmic miRNAs and lncRNAs (Supplementary Fig. 13).

These data support a dual-role model for *GbLINC-CNR1* (Fig. 4h). In males, it represses *GbAGL32a* to block ovule development and sequesters miR159 to maintain *GbMYB33* expression, supporting pollen formation. In females, absence of *GbLINC-CNR1* allows *GbAGL32a* to function, and miR159-mediated degradation of *GbMYB33* suppresses male pathways, channeling development toward ovule formation.

## Discussion

The sex determination mechanism of the ancient gymnosperm *G. biloba* has attracted considerable scientific interest. In contrast to angiosperms, which typically possess complex floral structures, gymnosperms like *Ginkgo* bear simple, unisexual strobili, and sexual differentiation occurs prior to gametogenesis. Earlier work established an XX/XY sex determination system in *Ginkgo*, dating the origin of its sex chromosomes to approximately 14 million years ago [3]. In this study, we employed BSA-seq to refine the location of the SDR to a 9 Mb interval (216.5-225.5 Mb) on chromosome 2. Within this region, the pattern of SNP genotypes—predominantly heterozygous in males and homozygous in females—reinforces the model of an XY system [3]. Our mapped interval aligns broadly with the ~ 50 Mb region defined by markers evm.chr2.564 and evm.chr2.722 reported by Gong et al. [37]., who used the same high-quality chromosome-level reference genome [2]. The discrepancy with the SDR location proposed by Liao et al. [3]. (~ 48–75 Mb on chromosome 2) likely stems from fundamental differences in the underlying datasets. The study by Liao et al. was based on an early, unpublished genome assembly [41], whereas subsequent work, including ours, has utilized the nearly complete reference [2].

MADS-box transcription factors are central to the development of reproductive structures in both angiosperms and gymnosperms. In flowering plants, various MADS-box genes have been directly linked to sex determination. For instance, in poplar, the sex-determining factor *ARR17* represses B-class *AP3* expression to suppress stamen formation [27], while in melon, *CmLHP1* proteins inhibit the expression of *CmWIP1* and the B-class gene *PI* to maintain female flower identity [42]. Within gymnosperms, differential expression of B-class *DEF/GLO* homologs is associated with male cone development, hinting that functional divergence of these genes could be a shared theme in seed plant sex determination [43]. In *Ginkgo*, a previous study identified *GbMADS18*, a GGM13-like (B-sister) gene expressed in male strobili [3]. Phylogenetically, this gene belongs to a lineage distinct from canonical B-class genes like *AP3/PI* and is more often involved in seed coat and ovule development [44]. In contrast, our research pinpointed *GbAP3a*—a clear ortholog of the classical B-class gene *AP3*—within the SDR. Its expression is strongly male-biased, being abundant in microstrobili and virtually absent in ovules. This sharp, binary expression pattern is a hallmark of a key regulatory switch governing divergent reproductive organ development [45]. Despite the current lack of stable genetic transformation for *Ginkgo* [46, 47], multiple lines of evidence collectively argue for *GbAP3a* as a primary determinant of male reproductive fate in *Ginkgo*.

AGL32 belongs to the B-sister clade, an ancient lineage of MADS-box genes that forms the sister group to the B-class floral homeotic genes (*AP3*/*PI*). This clade originated via gene duplication in the common ancestor of seed plants approximately 300 million years ago [48]. B-sister genes are present in both gymnosperms and angiosperms, and the phylogenomic analysis by Chen et al. (2017) [38] also identified *AGL32* as one of the MIKC^C^ clades retained in gymnosperms. In our study, *GbAGL32a* showed highly specific expression in female strobili and was almost undetectable in male strobili, a pattern that matches the evolutionarily conserved female‑biased expression characteristic of B-sister genes. Unlike B-class genes, which are predominantly expressed in male reproductive organs, B-sister genes are mainly transcribed in female reproductive organs, particularly in the integuments of ovules [48]. In *Arabidopsis*, *TT16/ABS* is required for endothelial cell differentiation and proanthocyanidin biosynthesis in the seed coat [44], and the *abs stk* double mutant exhibits defective inner integument development and aberrant ovule formation [49]. In rice, mutation of *FST* (an orthologue of B-sister) causes abnormal ovules and complete abortion of embryos and endosperm [50]. Protein interaction studies have shown that B-sister proteins can form multimeric complexes with SEP (E-class) and STK (D-class) MADS-box proteins [51]. In gymnosperms, the B-sister gene *GGM13* from *Gnetum gnemon* is expressed in the nucellus and the inner envelope of the female reproductive unit [48], and AGL32-like genes in *Cunninghamia lanceolata* are expressed in female cones, male cones, seeds, female gametophytes, and ovuliferous scales [52]. Collectively, these findings indicate that the role of B-sister genes in female reproductive development has been conserved across seed plants.

In our study, *GbAGL32a* clustered with *GbAGb*, *GbAGL6a/b*, and *GbSVPa* as a female-co-upregulated module. Considering that gymnosperms naturally lack E-class (SEP) genes, and that B-sister proteins are known to interact with SEP-like proteins [51], we propose that *GbAGL32a* may participate in alternative multimeric complexes with *GbAGb* and *GbAGL6a/b* to drive female reproductive organ specification in *Ginkgo*. This finding is consistent with the gymnosperm “BC system” framework, where C-class genes specify reproductive organ identity and B-class genes distinguish male from female organs [39, 43]. Within this framework, the B-sister gene *GbAGL32a* likely serves as an auxiliary factor that reinforces female reproductive development, potentially compensating for the absence of E-class proteins in gymnosperms. Our results thus provide new evidence for the conserved involvement of B-sister genes in female reproductive regulation across seed plants, while clarifying their auxiliary role within the simplified BC system of gymnosperms. However, definitive functional validation of *GbAGL32a* awaits the establishment of a reliable transformation system for *G. biloba*.

Long noncoding RNAs have emerged as versatile regulators in plant development, including reproduction. We identified *GbLINC-CNR1* that appears to orchestrate sex differentiation through two concurrent mechanisms. First, it directly targets the mRNA of the ovule-promoting gene *GbAGL32a*, leading to its repression and thereby preventing female organogenesis in male plants. Analogous lncRNA-mediated repression has been observed in poplar sex determination [9], suggesting this could be a conserved strategy among dioecious species. Second, *GbLINC-CNR1* acts as a molecular sponge for miR159. The miR159-MYB33 pathway is crucial for anther and pollen development [53, 54]. By sequestering miR159, *GbLINC-CNR1* protects *GbMYB33* transcripts from degradation, sustaining the expression needed for male reproductive development. The co-localization of the *GbMYB33* protein in the nucleus and cytoplasm supports the feasibility of this ceRNA model, as it suggests the corresponding mRNA is present in the cytoplasm, accessible to both miR159 and the lncRNA.

However, it is important to note the limitations of our current model. The direct interactions proposed—between *GbLINC-CNR1* and *GbAGL32a* mRNA, and between *GbLINC-CNR1* and miR159—are primarily supported by bioinformatic predictions and correlative expression data. The subcellular localization of *GbLINC-CNR1* itself, which would inform its mode of action, remains undetermined. Future work employing methods like RNA immunoprecipitation sequencing (RIP-seq) or degradome analysis is essential to validate these molecular interactions definitively.

## Conclusion

In summary, our work provides a refined map of the *Ginkgo* SDR and proposes a model where sex determination is driven by the functional divergence of B- and B-sister MADS-box genes, integrated with dual regulatory input from a sex-linked lncRNA. While requiring further functional validation, these findings significantly advance our understanding of sex determination in gymnosperms and highlight potential molecular targets for future research and breeding in dioecious tree species.

## Materials and methods

### Plant materials and morphological observation

We collected G. biloba samples from multiple sites in Nanjing, China, including the Nanjing Forestry University campus, Xuanwu Lake Park, and street trees. All trees were over 10 years old, healthy, and their sex had been confirmed over multiple flowering seasons. For BSA-seq, we pooled 30 male trees and 30 female trees separately. For transcriptome and lncRNA sequencing, we collected the same tissues from three male and three female trees as biological replicates. Samples were frozen in liquid nitrogen and stored at − 80 °C.Morphology of reproductive organs was observed across developmental stages: winter dormancy, spring bud break, and maturity. Images were captured with a digital camera.

### Whole-genome resequencing and genetic variation analysis

Genomic DNA was extracted from 30 female and 30 male samples using a plant DNA kit (Tiangen, DP320). Equal amounts of DNA from each sex were pooled to construct male and female libraries. DNA quality was assessed with a NanoDrop and agarose gels. Libraries were sequenced on an Illumina NovaSeq 6000 (150 bp paired‑end), aiming for ~ 60× depth per pool.

Raw reads were filtered using fastp v0.23.2 [55] by removing adapter-contaminated reads, reads containing more than 5% ambiguous nucleotides (N), and reads with over 50% bases having a Phred quality score below 20. High-quality reads were aligned to the Ginkgo reference genome [2] using BWA-MEM v0.7.17 [56] with default parameters. Alignment files were sorted using SAMtools v1.17 [57], and duplicate reads were marked using the Picard Toolkit v2.26.1 (http://broadinstitute.github.io/picard). GATK v4.2.6.1 [58] was employed for local realignment and base quality recalibration (BQSR) to call SNPs and InDels, following best-practice workflows. Variants were functionally annotated using ANNOVAR (2020Jun08 release) [59]. Genome-wide variant density was visualized using the R package CMplot v4.5.1 [60] with a 10 Mb non-overlapping sliding window. Linkage disequilibrium (LD) within the SDR was calculated using PLINK v1.90b6.21 [61] and visualized with LDBlockShow v1.40 [62] using default settings.

### Mapping sex-determination intervals using bulked segregant analysis

For BSA-seq analysis, raw SNPs were filtered by excluding loci with multiple genotypes, low depth (< 4×), or non-informative homozygous status in both pools. Euclidean Distance (ED) values between male and female allele frequencies were calculated as previously described [63]. To enhance the signal-to-noise ratio, ED values were raised to the fifth power (ED⁵). Genome-wide ED⁵ values were calculated using a sliding-window approach with a 5-Mb window size and 500-kb step size. Candidate SDRs were identified based on permutation-derived significance thresholds, and genomic intervals exceeding the 99% confidence threshold were considered significantly associated with sex determination.

### Transcriptome sequencing and differential expression analysis

We prepared strand‑specific libraries with the Illumina TruSeq Stranded mRNA Kit and sequenced them on a NovaSeq 6000. Raw reads were processed using Trimmomatic v0.39 [64] to remove adapter sequences, low-quality reads, and reads containing excessive ambiguous bases. Clean reads were aligned to the reference genome using STAR v2.7.10a [65] with default parameters. Gene-level read counts were quantified using featureCounts v2.0.3 [66]. Gene expression levels were normalized as fragments per kilobase of transcript per million mapped reads (FPKM). Biological replicate consistency was evaluated using Pearson correlation analysis and principal component analysis (PCA). Differentially expressed genes (DEGs) were identified using DESeq2 v1.34.0 [67]. P-values were adjusted using the Benjamini–Hochberg method, and genes with an adjusted p value < 0.05 and |log₂FC| ≥ 1 were considered significantly differentially expressed.

### Long noncoding RNA sequencing and bioinformatics analysis

Total RNA was extracted using TRIzol reagent (Thermo Fisher Scientific). Following rRNA depletion using the Ribo-Zero Gold Kit (Illumina), strand-specific libraries were constructed using the NEBNext Ultra II Directional RNA Library Prep Kit and sequenced on the NovaSeq 6000 platform. Clean reads were aligned to the reference genome using HISAT2 v2.2.1 [68] with default parameters, and transcript assembly was performed using StringTie2 v2.2.1 [69]. Candidate lncRNAs were retained only if they were longer than 200 nt and contained at least two exons. Coding potential was evaluated using CPC2 [70], CNCI v2.0 [71], LGC v1.0 [72], and the Pfam database v35.0 [73]. Only transcripts consistently classified as non-coding by all methods were retained. LncRNA types (lincRNA, intronic, antisense, sense) were assigned with Cuffcompare v2.2.1 [74]. Secondary structures were predicted using ViennaRNA v2.6.4 [75]. Differentially expressed lncRNAs (DELs) were defined using thresholds of adjusted p < 0.05 and |log₂FC| ≥ 1. Motif enrichment and functional annotation analyses were conducted using DREME v5.5.6 [76] and GOMo v5.5.6 [77], respectively. Cis-target genes were defined as protein-coding genes located within 100 kb upstream or downstream of each lncRNA, whereas trans-targets were predicted using LncTar v1.0 [78]. The ceRNA network was constructed using psRNATarget v2.0 (2017 release) [79] and miRBase Release 22.1 [80] and visualized in Cytoscape v3.9.1 [81].

### Functional, structural, and evolutionary analyses of sex-determining candidate genes

GO and KEGG enrichment analyses were performed using clusterProfiler v3.16 [82], with adjusted p values < 0.05 considered significant. MADS-box homologs were identified using BLASTP v2.12.0+ [83] (E-value ≤ 1e-10). Conserved domains were verified using Pfam [73] and CDD [84]. Genomic distributions were visualized using Circos v0.69-9 [85].

To determine the phylogenetic positions of the 23 newly identified Ginkgo MIKC-type MADS-box genes, full-length protein sequences were aligned using MAFFT v7.525 [86] with the AUTO strategy. Poorly aligned regions were removed using trimAl v1.5.rev1 [87] with the automated trimming mode. A maximum-likelihood phylogenetic tree was reconstructed using IQ-TREE v3.1.2 [88] with the Q.PLANT+R6 model [38] with 1,000 ultrafast bootstrap replicates, and the resulting tree was visualized using iTOL v6 [89]. Promoter regions (2 kb upstream) were analyzed for cis-elements with PlantPAN 4.0 [90, 90]. Sequence variants were visualized with Jalview v2.11.4.1 [91]. Gene duplication events were identified using MCScanX-transposed [92], and Ka/Ks ratios were calculated using KaKs_Calculator 2.0 [93] under the YN model [94].

### Subcellular localization analysis

Coding sequences of GbMYB33a and GbMYB33b (without stop codon) were cloned into pCAMBIA1302 to generate 35 S::GbMYB33-GFP fusions. A nuclear marker (35 S::RFP-NLS) was used. Constructs were transformed into Agrobacterium tumefaciens GV3101. Bacteria were cultured to OD600 = 0.8-1.0, harvested, and resuspended in infiltration buffer (10 mM MES, 10 mM MgCl2, 150 µM acetosyringone, pH 5.6). After 2–3 h in darkness, suspensions were infiltrated into leaves of 4–6 week‑old Nicotiana benthamiana. Plants were kept in darkness for 24 h, then under a 16/8 h light/dark cycle at 22 °C for 48 h. GFP and RFP fluorescence were imaged with a Leica TCS SP8 confocal microscope (GFP: 488 nm excitation, 500–540 nm emission; RFP: 561 nm excitation, 580–620 nm emission).

## Supplementary Information

Supplementary Material 1.

## Data Availability

The raw sequencing data generated in this study have been deposited in the Genome Sequence Archive (GSA) at the National Genomics Data Center (NGDC), China National Center for Bioinformation (CNCB), under BioProject accession number PRJCA047841. The whole-genome resequencing data for BSA-seq analysis are available under accession number CRA031283. The lncRNA sequencing data are accessible under accession number CRA031221, and the RNA-seq data are available under accession number CRA031210.

## Abbreviations

BSA-seq: Bulked Segregant Analysis sequencing
SDR: Sex-Determining Region
GbLINC-CNR1: Cellular Negative Regulator 1
LncRNAs: Long noncoding RNAs
eTMs: Endogenous Target Mimics
SNPs: Single Nucleotide Polymorphisms
InDel: Insertion/Deletion
ED: Euclidean Distance
GO: Gene Ontology
LD: Linkage disequilibrium
DEGs: Differentially Expressed Genes
DELs: Differentially Expressed LncRNAs
ceRNA: Competing endogenous RNA
