# Supplementary material for: Functional divergence of MADS-box genes and the dual regulatory role of a long noncoding RNA underlie sex determination in Ginkgo biloba
Source: BMC Plant Biol. 2026 Jul 10;26:1698. doi: 10.1186/s12870-026-09503-9 (PMC13640209; doi:10.1186/s12870-026-09503-9)
Supplement: Supplementary file 1 [file 12870_2026_9503_MOESM1_ESM.docx]

**Supplemental Figures**

**Supplementary Figure 1** Genome-wide distribution characteristics of SNPs and InDels in *G. biloba*.

Heatmaps display the chromosomal distribution of SNPs (left) and InDels (right) across all 12 chromosomes of *G. biloba*. Variant density was calculated using a 10 Mb non-overlapping sliding window. The color gradient transitions from blue (low density) to red (high density).

**Supplementary Figure 2** Sex-specific SNP genotypes within the SDR of *G. biloba*.

**(a)** Physical distribution of SNP genotypes in male and female SDRs.

**(b)** Proportion of homozygous and heterozygous genotypes in male and female SDRs.

**Supplementary Figure 3** GO enrichment analysis of genes within the SDR of *G. biloba*.

Bar plots depict significantly enriched GO terms across three major categories for 31 SDR genes: molecular function (MF; blue), cellular component (CC; green), and biological process (BP; red). The x-axis lists the enriched GO terms, and the y-axis indicates the number of annotated genes.

**Supplementary Figure 4** Identification and expression pattern analysis of differentially expressed genes between male and female *G. biloba*.

**(a)** Volcano plot of differentially expressed genes, where each point represents a gene. Red and green points indicate genes significantly upregulated or downregulated in male individuals, respectively, and grey points represent genes without significant differential expression.

**(b)** Distribution of gene expression levels. f1, f2, and f3 represent three biological replicates of female samples, and m1, m2, and m3 represent three biological replicates of male samples. Different colored segments within each bar correspond to distinct intervals of expression levels, which are presented as Fragments Per Kilobase of transcript per Million mapped reads (FPKM).

**Supplementary Figure 5** Functional enrichment analysis of DEGs in *G. biloba*.

**(a)** GO enrichment analysis of DEGs. Bar length represents the total number of genes enriched in each term, with the red and blue portions indicating the number of upregulated and downregulated genes, respectively.

**(b)** Kyoto Encyclopedia of Genes and Genomes (KEGG) pathway enrichment analysis of DEGs. Each bubble represents a significantly enriched pathway. Bubble size is proportional to the number of genes enriched in the pathway, and bubble color from yellow to blue indicates increasing enrichment significance. The Gene Ratio on the x-axis represents the proportion of DEGs in a pathway relative to the total number of DEGs that were annotated to the KEGG database.

**Supplementary Figure 6** Chromosomal distribution and syntenic relationships of MADS-box genes in the *G. biloba* genome.

The circos plot displays the genomic positions of GbMADS genes on the eight chromosomes containing these loci and summarizes their syntenic relationships. The outer ring marks the corresponding chromosome segments, and each GbMADS gene is mapped according to its physical coordinates. Gray links represent genome-wide collinearity, while green and blue links indicate tandem and proximal duplications involving GbMADS genes. Gene labels follow the same color scheme to highlight their association with specific duplication categories.

**Supplementary Figure 7** Phylogenetic reconstruction of MIKC-type MADS-box genes in *G. biloba* and reference seed plants.

The maximum-likelihood phylogenetic tree was reconstructed by incorporating the 23 newly identified MIKC full-length protein sequences from the *G. biloba* genome together with the reference MIKC sequences from representative gymnosperms and angiosperms, following the phylogenetic framework established by Chen *et al*. (2017). All *Ginkgo* sequences are highlighted in red for clear identification. Among them, genes with the prefix "Gb" were previously identified in *Ginkgo* by Chen *et al.* (2017), while genes with the prefix "chr" represent the newly identified MIKC^C^ members in this study.

**References:**

Chen, F., Zhang, X., Liu, X., & Zhang, L. (2017). Evolutionary analysis of MIKC^C^ -type MADS-box genes in gymnosperms and angiosperms. *Frontiers in Plant Science*, 8, 895.

**Supplementary Figure 8** Alignment of promoter sequences of the *GbAP3a* gene from female and male *G. biloba*.

*GbAP3a*-F and *GbAP3a*-M represent the *GbAP3a* gene promoter sequences from female and male individuals, respectively. Polymorphic sites between the female and male promoter sequences are highlighted in red.

**Supplementary Figure 9** Identification and classification of novel lncRNAs in *G. biloba*.

**(a)** The Venn diagram illustrates the filtering process for novel lncRNAs, in which candidate transcripts were assessed using PFAM (Protein Family database alignment), CNCI (Coding-Non-Coding Index), CPC2 (Coding Potential Calculator 2) and LGC (Long transcript assessment based on GC content and length).

**(b)** The bar chart presents the genomic classification of lncRNAs, including intergenic, intronic and antisense types.

**Supplementary Figure 10** Identification and expression patterns of differentially expressed lncRNAs.

**(a)** Volcano plot of differentially expressed lncRNAs between female and male samples. Red, blue, and gray dots represent lncRNAs that are significantly upregulated, downregulated, or not significantly changed, respectively, in male samples.

**(b)** Stacked bar chart of the number of DELs in each sample. The color segments correspond to distinct expression abundance categories defined by FPKM values.

**Supplementary Figure 11** Minimum free energy (MFE) secondary structure of *GbLINC-CNR1*.

The structure is color-coded by base-pairing probability on a scale from 0 (purple) to 1 (red), where red indicates the highest confidence.

**Supplementary Figure 12** Subcellular localization of *GbMYB33a* and *GbMYB33b*.

*GbMYB33a* and *GbMYB33b* were transiently expressed as GFP-fusion proteins in *Nicotiana benthamiana* leaves. Green fluorescence indicates the GFP signal from the GbMYB33-GFP fusion proteins. Red fluorescence marks the nucleus, visualized by a co-expressed RFP fused to a nuclear localization signal (NLS). Scale bar = 20 µm.

**Supplemental Tables**

**Supplementary Table 1** Summary of whole-genome resequencing data for BSA-seq pools of *G. biloba*.

| **Parameter** | **Female Pool** | **Male Pool** | **Total** |
| --- | --- | --- | --- |
| Number of raw reads | 3,792,367,778 | 3,931,080,330 | 7,723,448,108 |
| Total raw bases (Gb) | 568.86 | 589.66 | 1158.52 |
| Number of clean reads | 3,661,346,406 | 3,790,464,712 | 7,451,811,118 |
| Total clean bases (Gb) | 549.2 | 568.57 | 1117.77 |
| Clean read rate (%) | 96.55 | 96.42 | 96.49 |
| Q30 rate (%) | 92.45 | 92.56 | 91.51 |
| Mapping rate (%) | 91.56 | 90.85 | 91.23 |

**Supplementary Table 2** Chromosomal distribution and density of SNPs and InDels in the *G. biloba* genome.

| **Chromosome** | **Length**  **(Mb)** | **Number of SNPs** | **Number of InDels** | **SNP Density (SNPs/kb)** | **InDel Density (InDels/kb)** |
| --- | --- | --- | --- | --- | --- |
| chr1 | 1,185.86 | 8,446,801 | 444,447 | 7.12 | 0.37 |
| chr2 | 809.34 | 5,827,128 | 310,240 | 7.2 | 0.38 |
| chr3 | 783.85 | 5,476,560 | 279,659 | 6.99 | 0.36 |
| chr4 | 783.43 | 5,447,049 | 274,797 | 6.95 | 0.35 |
| chr5 | 762.93 | 5,449,032 | 261,005 | 7.14 | 0.34 |
| chr6 | 754.57 | 5,420,757 | 287,054 | 7.18 | 0.38 |
| chr7 | 747.81 | 5,117,108 | 269,185 | 6.84 | 0.36 |
| chr8 | 731.98 | 5,197,746 | 284,960 | 7.1 | 0.39 |
| chr9 | 728.9 | 5,139,783 | 525,036 | 7.05 | 0.72 |
| chr10 | 708.91 | 5,035,051 | 254,100 | 7.1 | 0.36 |
| chr11 | 669.79 | 4,770,759 | 242,669 | 7.12 | 0.36 |
| chr12 | 667.57 | 4,885,793 | 238,000 | 7.32 | 0.36 |
| Total | 9,334.94 | 66,213,567 | 3,671,152 | 7.09 | 0.39 |

**Supplementary Table 3** Functional annotation of SNP and InDel variants identified by BSA-seq in *G. biloba*.

| **Variant Category** | **Number of SNPs** | **Number of InDels** |
| --- | --- | --- |
| Intergenic | 63,390,718 | 4,438,044 |
| Intronic | 3,453,559 | 275,070 |
| Upstream (≤1 kb) | 112,576 | 22,857 |
| Downstream (≤1 kb) | 91,249 | 17,208 |
| Exonic (total) | 73,688 | 4,959 |
| Nonsynonymous SNV | 45,641 | – |
| Synonymous SNV | 26,655 | – |
| Stop-gain | 1,112 | 216 |
| Start-loss | 156 | 31 |
| Stop-loss | 121 | 61 |
| Unknown | 3 | 1 |
| Splice site region | 403 | 127 |
| Frameshift deletion | – | 1,620 |
| Frameshift insertion | – | 1,606 |
| Non-frameshift deletion | – | 807 |
| Non-frameshift insertion | – | 602 |
| 5′ UTR | – | 4 |
| Total | 67,122,193 | 4,762,904 |

**Supplementary Table 4** ED values from sliding window analysis across the putative SDR on chromosome 2 of *G. biloba*.

| **Chromosome** | **Window Range (Mb)** | **Window Center (Mb)** | **Mean ED ^a^** |
| --- | --- | --- | --- |
| chr2 | 214.0–219.0 | 216.5 | 0.059 |
| chr2 | 214.5–219.5 | 217 | 0.061 |
| chr2 | 215.0–220.0 | 217.5 | 0.063 |
| chr2 | 215.5–220.5 | 218 | 0.064 |
| chr2 | 216.0–221.0 | 218.5 | 0.068 |
| chr2 | 216.5–221.5 | 219 | 0.067 |
| chr2 | 217.0–222.0 | 219.5 | 0.069 |
| chr2 | 217.5–222.5 | 220 | 0.086 |
| chr2 | 218.0–223.0 | 220.5 | 0.097 |
| chr2 | 218.5–223.5 | 221 | 0.098 |
| **chr2** | **219.0–224.0** | **221.5** | **0.115** |
| **chr2** | **219.5–224.5** | **222** | **0.115** |
| **chr2** | **220.0–225.0** | **222.5** | **0.114** |
| chr2 | 220.5–225.5 | 223 | 0.112 |
| chr2 | 221.0–226.0 | 223.5 | 0.105 |
| chr2 | 221.5–226.5 | 224 | 0.098 |
| chr2 | 222.0–227.0 | 224.5 | 0.083 |
| chr2 | 222.5–227.5 | 225 | 0.077 |
| chr2 | 223.0–228.0 | 225.5 | 0.065 |

**Notes:** The analysis was conducted using a sliding window approach with a window size of 5 Mb and a step size of 500 kb. This table displays the results for the region surrounding the candidate SDR.

**^a^** Mean ED was calculated based on the allele frequency differences between the male and female pools for all SNPs within each window. The peak ED values, indicating the strongest association with the sex-determining locus, are highlighted in bold.

**Supplementary Table 5** ED values and functional annotation of 31 genes within the SDR on chromosome 2 of *G. biloba*.

| **ID** | **Gene ID** | **Gene Symbol** | **Genomic Position**  **(chr2, bp)** | **Gene  ED value ^a^** | **Functional Annotation ^b^** |
| --- | --- | --- | --- | --- | --- |
| 1 | chr2.619 | *GbCNR* | 216,962,968–216,963,612 | 0.061 | Cell number regulator |
| 2 | chr2.620 | *GbRRP* | 217,089,058–217,202,443 | 0.061 | Ribosomal RNA-processing protein |
| 3 | chr2.621 | N/A | 217,249,243–217,249,617 | 0.061 | Uncharacterized protein |
| 4 | chr2.622 | *GbRHa* | 217,348,826–217,351,293 | 0.063 | RNA helicase |
| 5 | chr2.623 | *GbRHb* | 217,407,927–217,408,688 | 0.063 | RNA helicase |
| 6 | chr2.624 | *GbRHc* | 217,495,040–217,497,522 | 0.063 | RNA helicase |
| 7 | chr2.625 | *GbRHd* | 217,940,867–217,942,103 | 0.064 | RNA helicase |
| 8 | chr2.626 | *GbRHe* | 218,427,935–218,430,143 | 0.068 | RNA helicase |
| 9 | chr2.627 | *GbRHf* | 218,488,226–218,490,430 | 0.068 | RNA helicase |
| 10 | chr2.628 | *GbNGBPa* | 218,587,407–218,588,796 | 0.068 | Nucleolar GTP-binding protein |
| 11 | chr2.629 | *GbPPRa* | 218,682,318–218,682,973 | 0.068 | Pentatricopeptide repeat-containing protein |
| 12 | chr2.630 | *GbNGBPb* | 218,685,075–218,687,087 | 0.068 | Nucleolar GTP-binding protein |
| 13 | chr2.631 | *GbLURPa* | 218,909,004–218,909,467 | 0.067 | Leucine-rich repeat and WD repeat-containing protein |
| 14 | chr2.632 | *GbLURPb* | 218,909,637–218,909,835 | 0.067 | Leucine-rich repeat and WD repeat-containing protein |
| 15 | chr2.633 | *GbRRa* | 219,238,969–219,239,459 | 0.067 | Two-component response regulator |
| 16 | chr2.634 | N/A | 219,655,638–219,656,725 | 0.069 | Uncharacterized protein |
| 17 | chr2.635 | *GbUNI* | 220,061,212–220,062,503 | 0.086 | Disease resistance protein |
| 18 | chr2.636 | *GbFGGY* | 220,175,449–220,385,759 | 0.086 | FGGY carbohydrate kinase domain-containing protein |
| 19 | chr2.637 | *GbBIO3a* | 220,687,629–220,880,410 | 0.097 | Bifunctional DTBS/DAPA aminotransferase |
| 20 | chr2.638 | *GbBIO3b* | 220,985,472–220,986,907 | 0.098 | Bifunctional DTBS/DAPA aminotransferase |
| 21 | **chr2.639** | ***GbAP3b*** | **221,458,611–221,460,820** | **0.115** | **MADS-box protein** |
| 22 | **chr2.640** | ***GbAP3c*** | **221,705,055–221,706,817** | **0.115** | **MADS-box protein** |
| 23 | **chr2.641** | ***GbAP3a*** | **222,135,927–222,138,356** | **0.115** | **MADS-box protein** |
| 24 | chr2.642 | *GbNUFD* | 222,545,795–222,548,647 | 0.114 | Nuclear fusion defective protein |
| 25 | chr2.643 | *GbABH* | 222,974,009–222,975,390 | 0.112 | Alpha/beta hydrolase domain-containing protein |
| 26 | chr2.644 | *GbINO80* | 223,526,238–223,529,807 | 0.105 | INO80 complex subunit B-like conserved region |
| 27 | chr2.645 | *GbRRb* | 223,874,331–223,903,763 | 0.098 | Two-component response regulator |
| 28 | chr2.646 | *GbLSD* | 224,373,186–224,379,098 | 0.083 | Lysine-specific demethylase |
| 29 | chr2.647 | N/A | 224,494,913–224,497,060 | 0.083 | Uncharacterized protein |
| 30 | chr2.648 | *GbBAT* | 224,876,391–224,879,158 | 0.083 | Brassinosteroid-related acyltransferase |
| 31 | chr2.649 | *GbPPRb* | 225,104,852–225,283,355 | 0.077 | Pentatricopeptide repeat-containing protein |

**Notes:**

**^a^** Gene ED value was calculated as the average Euclidean Distance of all SNPs located within the genomic coordinates of each gene.

**^b^** Genes highlighted in bold are located at the core ED peak and showed significant enrichment in GO terms related to flower development.

**Supplementary Table 6** Summary statistics of RNA-seq data from *G. biloba* male and female samples.

| **Sample ID** | **Raw**  **Reads** | **Raw Bases (Gb)** | **Clean Reads** | **Clean Bases**  **(Gb)** | **Clean Read Rate (%)** | **Q30 (%)** ^c^ |
| --- | --- | --- | --- | --- | --- | --- |
| f1 ^a^ | 75,087,686 | 11.26 | 72,424,230 | 10.86 | 96.45 | 94.17 |
| f2 | 80,716,456 | 12.11 | 78,086,616 | 11.71 | 96.74 | 94.34 |
| f3 | 80,072,970 | 12.01 | 77,441,386 | 11.62 | 96.71 | 94.2 |
| m1 ^b^ | 80,054,254 | 12.01 | 76,354,580 | 11.45 | 95.38 | 94.05 |
| m2 | 77,864,578 | 11.68 | 75,157,978 | 11.27 | 96.52 | 94.04 |
| m3 | 70,066,068 | 10.51 | 67,804,618 | 10.17 | 96.77 | 94.2 |

**Notes:**

^a^ f1, f2, and f3 represent three biological replicates of female *G. biloba* samples.

^b^ m1, m2, and m3 represent three biological replicates of male *G. biloba* samples.

^c^ Q30 indicates the percentage of bases with a Phred quality score ≥ 30.

**Supplementary Table 7** Characteristics of MADS-box genes in *G. biloba*.

| **Gene ID** | **Protein Length (aa)** | **Subfamily** |
| --- | --- | --- |
| chr1.173 | 452 | Type I |
| chr1.308 | 68 | Type II |
| chr1.1363 | 62 | Type II |
| chr1.1370 | 108 | Type II |
| chr1.1372 | 62 | Type II |
| chr1.1374 | 92 | Type I |
| chr1.1859 | 446 | Type I |
| chr3.869 | 210 | Type I |
| chr6.181 | 142 | Type II |
| chr6.184 | 65 | Type II |
| chr7.670 | 63 | Type II |
| chr7.1187 | 187 | Type II |
| chr10.1419 | 357 | Type I |
| chr11.1310 | 121 | Type II |
| chr12.1873 | 170 | Type I |
| chr1.1358 | 253 | Type II |
| chr1.1364 | 246 | Type II |
| chr1.1375 | 245 | Type II |
| chr1.1376 | 226 | Type II |
| chr1.1377 | 249 | Type II |
| chr1.1378 | 155 | Type II |
| chr2.639 | 258 | Type II |
| chr2.640 | 222 | Type II |
| chr2.641 | 235 | Type II |
| chr6.784 | 232 | Type II |
| chr7.1200 | 230 | Type II |
| chr11.906 | 278 | Type II |
| chr12.940 | 153 | Type II |
| chr12.1550 | 228 | Type II |

**Note**: Alternative Names are based on the putative orthologs in *Arabidopsis thaliana* identified by BLAST search and phylogenetic analysis. Protein Length is given in amino acids (aa). Subfamily classification into Type I and Type II was determined based on phylogenetic analysis. A hyphen (-) indicates that no clear ortholog was identified.

**Supplementary Table 8** Analysis of nonsynonymous (*Ka*) and synonymous (*Ks*) substitution rates for duplicated MADS-box gene pairs in *G. biloba*.

| **Gene 1** | **Gene 2** | ***Ka*** | ***Ks*** | ***Ka/Ks*** | **Duplication Mode** |
| --- | --- | --- | --- | --- | --- |
| *chr1.1374* | *chr1.1375* | 0.134465 | 0.156878 | 0.857129 | tandem |
| *chr1.1375* | *chr1.1376* | 0.106407 | 0.217295 | 0.489691 | tandem |
| *chr1.1376* | *chr1.1377* | 0.129128 | 0.338212 | 0.381795 | tandem |
| *chr1.1377* | *chr1.1378* | 0.313603 | 0.993276 | 0.315726 | tandem |
| *chr2.639* | *chr2.640* | 0.33563 | 0.883176 | 0.380025 | tandem |
| *chr2.640* | *chr2.641* | 0.301934 | 1.33209 | 0.226661 | tandem |
| *chr1.1363* | *chr1.1370* | 0.0729449 | 0.611007 | 0.119385 | proximal |
| *chr1.1370* | *chr1.1372* | 0.0555265 | 0.208806 | 0.265924 | proximal |

**Supplementary Table 9** Clade assignment and corresponding gene nomenclature for the 23 MIKC-type MADS-box genes identified in the *G. biloba* genome.

| **gene ID** | **MADS-box clade** | **Gene name**  **(this study)** | **Gene name**  **(Chen et al., 2017)** |
| --- | --- | --- | --- |
| chr7.1187 | AG | GbAGa |  |
| chr7.1200 | AG | GbAGb | Gb16301 |
| chr6.784 | AGL32 | GbAGL32a |  |
| chr11.1310 | AGL32 | GbAGL32b |  |
| chr6.184 | AGL6 | GbAGL6a |  |
| chr1.1358 | AGL6 | GbAGL6b | Gb36364 |
| chr1.1364 | AGL6 | GbAGL6c | Gb41549 |
| chr1.308 | ANR1 | GbANR1 |  |
| chr2.641 | AP3/PI | GbAP3a |  |
| chr2.639 | AP3/PI | GbAP3b | Gb28587 |
| chr2.640 | AP3/PI | GbAP3c |  |
| chr11.906 | AP3/PI | GbAP3d | Gb15398 |
| chr1.1363 | GMADS | GbGMADSa |  |
| chr1.1370 | GMADS | GbGMADSb | Gb01884 |
| chr1.1372 | GMADS | GbGMADSc |  |
| chr1.1375 | GMADS | GbGMADSd | Gb30604 |
| chr1.1376 | GMADS | GbGMADSe |  |
| chr1.1377 | GMADS | GbGMADSf | Gb19178 |
| chr1.1378 | GMADS | GbGMADSg | Gb39109 |
| chr6.181 | SOC1 | GbSOC1 |  |
| chr12.940 | SVP | GbSVPa |  |
| chr7.670 | SVP | GbSVPb | Gb05128 |
| chr12.1550 | MIKC* | chr12.1550 |  |

**Note:** Clade assignment was performed by phylogenetic reconstruction following the framework of Chen *et al*. (2017), which resolved MIKC-type MADS-box genes into 14 monophyletic clades based on genome and transcriptome data from all orders of gymnosperms and basal angiosperms.

Chen F, Zhang X, Liu X, Zhang L. Evolutionary analysis of MIKC^C^-type MADS-box genes in gymnosperms and angiosperms. *Front Plant Sci*. 2017; 8, 895.

**Supplementary Table 10** Differential expression analysis of ABCDE-class gene floral developmental genes homologs in male and female *G. biloba*.

| **Class** | **Gene ID** | **Gene Name** | **Female (FPKM)** | | | **Male (FPKM)** | | | **log₂FC** | **Adjusted** | **Regulation** |
| --- | --- | --- | --- | --- | --- | --- | --- | --- | --- | --- | --- |
|  |  |  | **f1** | **f2** | **f3** | **m1** | **m2** | **m3** | **(M/F)** | ***p*-value** |  |
| AG-C | **chr7.1200** | **GbAGb** | **322.8** | **559.9** | **443.7** | **132.8** | **115.7** | **192.8** | **-1.59** | **0.04** | **Down** |
|  | chr7.1187 | GbAGa | 0 | 0 | 0 | 0 | 0 | 0 | - | - | Not Sig. |
| AGL32 | **chr6.784** | **GbAGL32a** | **10.8** | **13.2** | **13.4** | **0.5** | **0.6** | **0.9** | **-4.24** | **0.0046** | **Down** |
|  | chr11.1310 | GbAGL32b | 0 | 0 | 0 | 0 | 0 | 0 | - | - | Not Sig. |
| AGL6 | **chr6.184** | **GbAGL6a** | **1342** | **1445** | **1826** | **209.3** | **260.9** | **338.8** | **-2.51** | **0.01** | **Down** |
|  | **chr1.1358** | **GbAGL6b** | **91.2** | **75.8** | **122.8** | **32.3** | **29.5** | **33.2** | **-1.61** | **0.04** | **Down** |
|  | chr1.1364 | GbAGL6c | 38.6 | 56.9 | 28.2 | 28.1 | 28.4 | 27.4 | -0.56 | 0.25 | Not Sig. |
| ANR1 | chr1.308 | GbANR1 | 0 | 0 | 0 | 0 | 0 | 0 | - | - | Not Sig. |
| AP3/PI-B | **chr2.641** | **GbAP3a** | **0** | **0.1** | **0** | **95** | **100.1** | **99.3** | **10.76** | **0.0003** | **Up** |
|  | chr2.639 | GbAP3b | 0 | 0.1 | 0 | 0.1 | 0 | 0.3 | 1.62 | 0.41 | Not Sig. |
|  | chr2.640 | GbAP3c | 0.2 | 0.1 | 2.4 | 0.2 | 0 | 0 | -3.87 | 0.4 | Not Sig. |
|  | chr11.906 | GbAP3d | 1.1 | 1.8 | 3.6 | 14.3 | 34.3 | 25.4 | 3.51 | 0.06 | Not Sig. |
| GMADS | **chr1.1376** | **GbGMADSe** | **2.3** | **1.5** | **1.9** | **1.3** | **0.7** | **0.5** | **-1.23** | **0.03** | **Down** |
|  | chr1.1363 | GbGMADSa | 8.3 | 13.9 | 7.7 | 3.7 | 3.1 | 2.8 | -1.64 | 0.07 | Not Sig. |
|  | chr1.1370 | GbGMADSb | 0 | 37.7 | 5.6 | 10.4 | 14.1 | 0 | -0.82 | 0.66 | Not Sig. |
|  | chr1.1372 | GbGMADSc | 0 | 4.5 | 0 | 0 | 1.7 | 0 | -1.38 | 0.61 | Not Sig. |
|  | chr1.1375 | GbGMADSd | 8.3 | 5.3 | 10.7 | 2 | 1.5 | 1 | -2.45 | 0.05 | Not Sig. |
|  | chr1.1377 | GbGMADSf | 1.5 | 1.1 | 0.9 | 1.3 | 0.7 | 1.7 | 0.05 | 0.9 | Not Sig. |
|  | chr1.1378 | GbGMADSg | 3.4 | 3 | 3.3 | 2 | 1.9 | 2 | -0.7 | 0.01 | Not Sig. |
| SOC1 | chr6.181 | GbSOC1 | 4.6 | 2.6 | 4.3 | 5.4 | 0.9 | 0.1 | -0.84 | 0.42 | Not Sig. |
| SVP | **chr12.940** | **GbSVPa** | **54.2** | **27.8** | **45.9** | **4.7** | **0.5** | **1.2** | **-4.34** | **0.03** | **Down** |
|  | chr7.670 | GbSVPb | 38.9 | 26.2 | 70.8 | 10.8 | 14 | 2.1 | -2.33 | 0.1 | Not Sig. |
| MIKC* | chr12.1550 | chr12.1550 | 0 | 0 | 0.1 | 3.2 | 0.4 | 0.7 | 5.93 | 0.25 | Not Sig. |

**Note:** Transcript abundances are expressed as Fragments Per Kilobase of transcript per Million mapped reads (FPKM). f1, f2, f3 are three biological replicates of female samples; m1, m2, m3 are three biological replicates of male samples. Differential expression was assessed by two-tailed Student’s t-test. Log_2_FC = log_2_(male FPKM / female FPKM). Genes with | Log_2_FC| ≥ 1 and Adjusted *p*-value < 0.05 were considered significantly differentially expressed. "Up" indicates significant upregulation in male samples; "Down" indicates significant downregulation; "Not Sig." indicates not significant.

**Supplementary Table 11** *Cis*-regulatory elements identified in the 2 kb promoter region of *GbAP3a* in *G. biloba*.

| ***Cis*-element name** | **Consensus Sequence** | **Count in Female** | **Count in Male** | **Function** |
| --- | --- | --- | --- | --- |
| **CArG** |  |  |  |  |
| CARGATCONSENSUS | CCWWWWWWGG | 2 | 2 | CArG box; Binding site for MADS-box TFs (AGL15, AGAMOUS), for floral organ identity |
| CARGCW8GAT | CWWWWWWWWG | 4 | 4 | CArG box; Binding site for MADS-box TFs (SOC1, FLC), involved in flowering regulation. |
| **Other** |  |  |  |  |
| DPBFCOREDCDC3 | ACACNNG | 4 | 4 | Seed maturation and ABA-responsive regulation (DPBF, bZIP, ABI5) |
| GT1GMSCAM4 | GAAAAA | 4 | 4 | Light-responsive GT-1 motif |
| ROOTMOTIFTAPOX1 | ATATT | 22 | 21 | Root-specific expression element |
| MYCATERD1 | CATGTG | 3 | 3 | Water-stress responsive MYC-binding site |
| CANBNNAPA | CNAACAC | 1 | 1 | Seed storage protein regulation (napA) |
| ACGTATERD1 | ACGT | 2 | 2 | ACGT motif for stress and etiolation response |
| ANAERO1CONSENSUS | AAACAAA | 2 | 1 | Anaerobic-responsive element |
| ARR1AT | NGATT | 26 | 25 | Cytokinin signaling via ARR1 binding site |
| GATABOX | GATA | 21 | 22 | Light GATA motif in chlorophyll and leaf |
| CAATBOX1 | CAAT | 24 | 23 | Promoter CAAT-box for seed expression |
| CURECORECR | GTAC | 12 | 10 | Copper and hypoxic stress-responsive element |
| SITEIIATCYTC | TGGGCY | 2 | 2 | Meristem and cytochrome via TCP-domain binding |
| MYBATRD22 | CTAACCA | 1 | 1 | ABA and water-stress responsive MYB-binding |
| DOFCOREZM | AAAG | 31 | 31 | DOF transcription factor binding site(leaf/shoot) |
| GTGANTG10 | GTGA | 15 | 14 | Pollen-specific GTGA motif |
| AACACOREOSGLUB1 | AACAAAC | 1 | 1 | Seed-specific glutelin and endosperm regulation |
| GT1CONSENSUS | GRWAAW | 15 | 17 | Light-responsive GT-1 consensus site |
| IBOXCORE | GATAA | 5 | 6 | I-box light-responsive element in photosynthetic |
| TAAAGSTKST1 | TAAAG | 11 | 11 | Guard cell-specific Dof binding site |
| OSE2ROOTNODULE | CTCTT | 5 | 3 | Root nodule-specific expression element |
| OSE1ROOTNODULE | AAAGAT | 4 | 5 | Root nodule-specific expression element |
| CACTFTPPCA1 | YACT | 33 | 31 | Mesophyll-specific promoter element |
| MYBCORE | CNGTTR | 4 | 4 | MYB-binding for stress and flavonoid biosynthesis |
| MYBST1 | GGATA | 2 | 2 | MYB-binding site |
| MYB2CONSENSUSAT | YAACKG | 2 | 2 | ABA-responsive MYB element |
| MYB1AT | WAACCA | 3 | 3 | ABA-responsive MYB element |
| EBOXBNNAPA | CANNTG | 20 | 18 | Seed storage protein E-box element |
| NAPINMOTIFBN | TACACAT | 1 | 1 | Napin seed storage protein motif |
| NODCON2GM | CTCTT | 5 | 3 | Nodulin-related root expression |
| NODCON1GM | AAAGAT | 4 | 5 | Nodulin-related root expression |
| WBOXATNPR1 | TTGAC | 2 | 2 | Defense-related WRKY-binding W-box |
| BOXIINTPATPB | ATAGAA | 3 | 3 | Plastid gene expression element (NEP promoter) |
| POLLEN1LELAT52 | AGAAA | 8 | 7 | Pollen-specific LAT52 motif |
| POLASIG2 | AATTAAA | 3 | 3 | Polyadenylation signal |
| POLASIG1 | AATAAA | 6 | 6 | Polyadenylation signal |
| POLASIG3 | AATAAT | 7 | 7 | Polyadenylation signal |
| -10PEHVPSBD | TATTCT | 4 | 4 | Light and circadian-regulated psbD promoter |
| S1FBOXSORPS1L21 | ATGGTA | 2 | 2 | Plastid ribosomal protein gene regulation |
| XYLAT | ACAAAGAA | 1 | 1 | Secondary xylem/wood formation element |
| SEF3MOTIFGM | AACCCA | 2 | 2 | Seed-specific 7S globulin regulation |
| SEF4MOTIFGM7S | RTTTTTR | 2 | 2 | Seed-specific storage protein regulation |
| WBOXHVISO1 | TGACT | 3 | 3 | Sugar- and WRKY-mediated stress response |
| TATABOX5 | TTATTT | 8 | 8 | Core promoter TATA-box |
| TATABOX4 | TATATAA | 4 | 3 | Core promoter TATA-box |
| TATAPVTRNALEU | TTTATATA | 3 | 2 | Core promoter TATA-box |
| ACGTTBOX | AACGTT | 2 | 2 | T-box transcription factor binding site |
| TATABOXOSPAL | TATTTAA | 2 | 2 | TATA-box with TBP/TFIIB binding |
| WBOXNTERF3 | TGACY | 4 | 4 | Wounding-responsive W-box |
| WBBOXPCWRKY1 | TTTGACY | 2 | 2 | WRKY-binding W-box |
| WRKY71OS | TGAC | 6 | 6 | WRKY71 binding site in GA and defense |
| -300ELEMENT | TGHAAARK | 5 | 4 | Seed storage protein regulation |
| LECPLEACS2 | TAAAATAT | 1 | 1 | Ethylene-responsive cysteine protease promoter |
| CPBCSPOR | TATTAG | 3 | 3 | Cytokinin- and chlorophyll-related element |
| RAV1AAT | CAACA | 14 | 15 | RAV1/AP2-B3 binding site in root and shoot |
| NTBBF1ARROLB | ACTTTA | 2 | 2 | Auxin-responsive Dof binding site in root/vascular |
| EECCRCAH1 | GANTTNC | 2 | 2 | Low-CO₂ responsive element |
| LEAFYATAG | CCAATGT | 1 | 1 | Floral meristem regulation (LEAFY, AGAMOUS) |
| CCAATBOX1 | CCAAT | 5 | 5 | CCAAT-box with heat shock responsiveness |
| ERELEE4 | AWTTCAAA | 1 | 1 | Ethylene-responsive element in fruit ripening |
| TATABOX2 | TATAAAT | 3 | 3 | Core promoter TATA-box |
| GT1CORE | GGTTAA | 1 | 1 | Light-responsive GT-1 element |
| SORLIP1AT | GCCAC | 1 | 2 | Light-inducible SORLIP1 motif |
| MYBCOREATCYCB1 | AACGG | 1 | 1 | Cell cycle regulation via MYB-binding site |
| MARARS | WTTTATRTTTW | 1 | 1 | Matrix/scaffold attachment region element |
| ARFAT | TGTCTC | 1 | 1 | Auxin-responsive element (AuxRE, ARF) |

**Supplementary Table 12** Summary of lncRNA sequencing data from male and female samples of *G. biloba*.

| **Sample ID** | **Raw Reads** | **Raw Bases (Gb)** | **Clean Reads** | **Clean Bases (Gb)** | **Clean Read Rate (%)** | **Q30 (%)** |
| --- | --- | --- | --- | --- | --- | --- |
| f1 | 184,285,754 | 27.64 | 148,698,322 | 22.3 | 80.69 | 91.47 |
| f2 | 149,984,886 | 22.5 | 93,441,486 | 14.01 | 62.3 | 90.1 |
| f3 | 147,003,936 | 26.1 | 120,399,190 | 18.06 | 69.19 | 90.91 |
| m1 | 172,478,064 | 25.87 | 137,287,308 | 20.59 | 79.6 | 94 |
| m2 | 153,132,118 | 22.97 | 118,553,222 | 17.78 | 77.42 | 91.17 |
| m3 | 182,554,658 | 27.38 | 134,958,200 | 20.24 | 73.93 | 93.52 |
| **Total / Average** | **989,439,416** | **152.46** | **753,337,728** | **112.98** | **73.86** | **91.86** |

**Note:** Samples f1–f3 and m1–m3 correspond to three biological replicates from female and male individuals, respectively.

**Supplementary Table 13** Enriched motifs of differentially expressed lncRNAs and their associated functional annotations.

| **Motif ID** | **Motif Sequence** | **Motif E-value** | **Motif Type** | **GO Term** | **GO ID** | ***P*-value** |
| --- | --- | --- | --- | --- | --- | --- |
| 1 | CTCKSC | 3.2E-6 | Transcription Factor Binding Site (TFBS) | CC: mitochondrion | GO:0005739 | 2.65E-7 |
|  |  |  |  | MF: transcription factor activity | GO:0003700 | 5.30E-7 |
|  |  |  |  | CC: chloroplast stroma | GO:0009570 | 1.59E-6 |
|  |  |  |  | BP: translation | GO:0006412 | 3.24E-5 |
|  |  |  |  | BP: stomatal complex morphogenesis | GO:0010103 | 3.29E-5 |
| 2 | TGGAGGW | 9.8E-3 | Not determined | CC: chloroplast stroma | GO:0009570 | 1.14E-5 |
| 3 | AGAGRAG | 2.7E-2 | Transcription Factor Binding Site (TFBS) | CC: chloroplast | GO:0009507 | 2.65E-7 |
|  |  |  |  | MF: transcription factor activity | GO:0003700 | 9.02E-6 |
|  |  |  |  | CC: nucleus | GO:0005634 | 9.28E-6 |
| 4 | ACACASAG | 4.2E-2 | Transcription Factor Binding Site (TFBS) | MF: transcription factor activity | GO:0003700 | 2.65E-7 |

**Note:** E-value represents the statistical significance of the motif enrichment. P-value corresponds to the significance of GO term enrichment. GO terms are categorized into Biological Process (BP), Molecular Function (MF), and Cellular Component (CC).
